# 2205. Potential Causative Association between Respiratory Viruses and Pneumococcus-Associated Disease in Young Children in Israel: Lessons from the COVID-19 Pandemic

**DOI:** 10.1093/ofid/ofac492.1824

**Published:** 2022-12-15

**Authors:** Ron Dagan, Bert Adriaan van der Beek, David Greenberg, Yonat Shemer Avni, Shalom Ben-Shimol, Daniel M Weinberger

**Affiliations:** Ben-Gurion University of the Negev, Beer Sheva, HaDarom, Israel; Ben Gurion Univerisity, Beer Sheva, HaDarom, Israel; Soroka University Medical Center, Beer Sheva, HaDarom, Israel; Soroka Medical Center, Beer Sheva, HaDarom, Israel; Soroka University Medical Center, Beer Sheva, HaDarom, Israel; Yale School of Public Health, New Haven, Connecticut

## Abstract

**Background:**

During the early Covid-19 pandemic, we observed a close-to-full disappearance of the activity of 4 respiratory viruses (RSV, hMPV, influenza, and parainfluenza), followed by an off-season sequential re-emergence in 2021. Surprisingly, a striking similarity between the dynamics of pneumococcus-associated disease (PAD; namely community-acquired alveolar pneumonia [CAAP; often considered pneumococcal] and bacteremic-pneumococcal pneumonia [IPD-Pneumonia]), was also observed. In contrast, adenovirus and rhinovirus activities did not change during COVID-19. We examined the association between PAD and RSV, hMPV, influenza, and parainfluenza (PAD-viruses).

**Methods:**

Surveillance of CAAP and IPD-Pneumonia incidences and viral activity in children < 5 years was described in detail previously [Danino D. et al. *Clin Infect Dis.* 2022, https://doi.org/10.1093/cid/ciab1014]. We extended the observations until December 2021, to capture the sequential re-emergence of the 4 PAD-viruses. A hierarchical linear regression model was used to quantify the association between PAD-viruses (each virus individually and combined), adenovirus and PAD. After fitting the models, the contribution of each virus was estimated.

**Results:**

The **Figure** shows striking similarities in the dynamics of CAAP, IPD-Pneumonia, and PAD-viruses both before and during the COVID-19 pandemic. During the expected peak season (Oct 2020 – Apr 2021) PAD episodes were extremely low. However, off-season peaks were seen during May – Dec 2021. Overall, 78% and 25% of all CAAP and IPD-Pneumonia episodes, respectively, were attributable to these viruses in children < 5 (**Table**). In CAAP, cases were attributable to each of the 4 PAD-viruses individually throughout the first 5 years of life: RSV and hMPV combined contributed 80%, 63%, and 42% of all CAAP episodes in children aged < 1, 1, and 2-4 years, respectively. The respective figures for influenza and parainfluenza combined were 13%, 21%, and 22%. Only RSV significantly contributed to IPD-Pneumonia (19%). Adenovirus did not contribute to PAD episodes.

**Figure:**
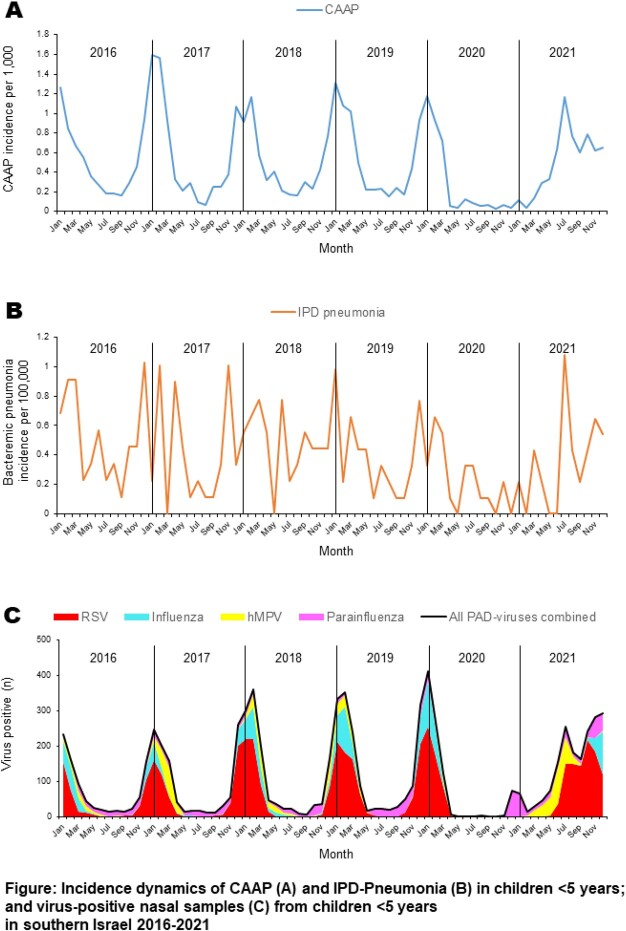

**Figure:**
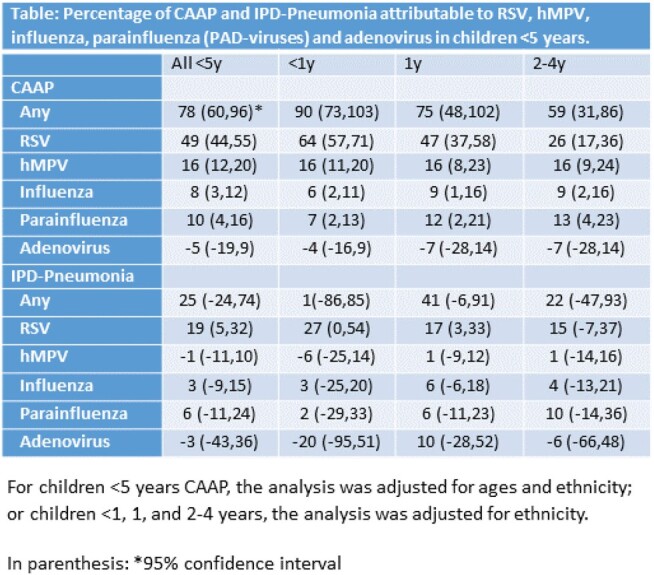

**Conclusion:**

Our model suggests an important causative association between RSV, hMPV, influenza, and parainfluenza viruses and CAAP, and between RSV and IPD-Pneumonia.

**Disclosures:**

**All Authors**: No reported disclosures.

